# Dietary nutrient intake and cancer presence: evidence from a cross-sectional study

**DOI:** 10.3389/fnut.2025.1551822

**Published:** 2025-04-01

**Authors:** Youjia Qin, Liu Chen, Zilong Zhao, Yuguan Li, Xuan Tian, Mingqian Feng, Jing Tang, Kangkang Ji

**Affiliations:** ^1^College of Life Science and Technology, Huazhong Agricultural University, Wuhan, Hubei, China; ^2^Department of Lymphoma, Hubei Cancer Hospital, Tongji Medical College, Huazhong University of Science and Technology, Wuhan, Hubei, China; ^3^College of Biomedicine and Health, Huazhong Agricultural University, Wuhan, Hubei, China; ^4^Department of Clinical Medical Research, Binhai County People’s Hospital, Clinical Medical College of Yangzhou University, Yancheng, Jiangsu, China

**Keywords:** cancer, solid cancer, blood cancer, dietary nutrient intake, NHANES

## Abstract

**Background:**

While the role of specific nutrients in cancer is established, associations between comprehensive between dietary nutrient intake and cancer presence remain underexplored. This cross-sectional study investigates global dietary nutrient profiles in relation to solid and blood cancers.

**Methods:**

A total of 42,732 mobile adults from the National Health and Nutrition Examination Survey (NHANES, 2001–2023) were enrolled in this study. The potential associations of dietary intakes of 34 nutrients and 4 common trace components with cancer presence were investigated by weighted logistic regression and restricted cubic spline.

**Results:**

Higher intake of saturated fatty acid (OR = 1.1082, 95% CI: 1.0110–1.2146), *β*-carotene (OR = 1.0431, 1.0096–1.0777) and vitamin K (OR = 1.0370, 1.0094–1.0654) was positively associated with overall cancer presence, while phosphorus intake (OR = 0.9016, 0.8218–0.9892) showed a protective association. For solid tumors, dietary intakes of saturated fatty acid (OR = 1.1099), *α*-carotene (OR = 1.0353), *β*-carotene (OR = 1.0484), and vitamin K (OR = 1.0405) exhibited positive associations. Retinol intake was linked to blood carcinoma (OR = 1.0935, 1.0222–1.1698). Dose–response analyses revealed linear relationships without non-linear thresholds.

**Conclusion:**

Specific dietary nutrients, notably saturated fats, carotenoids, and vitamin K, are associated with increased cancer presence, whereas phosphorus intake is associated with the reduced cancer presence. Due to the cross-sectional nature of the study and the measurement of dietary intake after cancer diagnosis, a causal relationship could not be established. These findings underscore the need for longitudinal studies to establish causality and inform dietary interventions in cancer management.

## Introduction

Cancer is the second leading cause of death on a global scale (1), with projections indicating a rising cancer-related disease burden over the coming decades (2). In 2024, the United States anticipates 2,001,140 new cancer cases and 611,720 deaths (3). By 2040, annual global cases may reach 29.9 million, with 15.3 million fatalities (4). Despite therapeutic advances, cancer biology remains complex (5), and the tumor microenvironment dynamically supports progression (6), complicating treatment. Diet critically influences tumor metabolism (7, 8), as nutrient adjustments alter tumor resource availability (9). Cancer cells adapt to nutrient scarcity by disrupting host homeostasis (10). Nutrient-deprivation therapies show broad efficacy (11–14), but benefits are short-lived due to tumor metabolic plasticity—for example, recruiting nerves to sustain growth under nutrient stress (14).

Solid tumors (e.g., breast, colorectal cancers) exploit stromal interactions and angiogenesis in nutrient-poor settings (14, 15). Conversely, blood cancers (e.g., leukemia, lymphoma) utilize systemic nutrients via bone marrow and circulation (16). Leukemia cells depend on lipid metabolism (17), while solid tumors reprogram glucose/glutamine pathways (18). These differences suggest cancer-specific nutritional strategies. Importantly, both cancer types share oxidative stress modulation and metabolic flexibility during dietary changes (19). Saturated fatty acids drive progression in solid tumors (breast, prostate, and colorectal) (20) and leukemia (21), urging cross-category dietary studies. Thus, understanding how combined nutrients regulate cancers is a translational priority.

Studies have investigated isolated nutrients: dietary fiber (22, 23), fatty acids (20, 24–26), β-carotene (27, 28), vitamin D (29–31), vitamin K (32, 33), caffeine (34, 35), selenium (36, 37), and others (38–40). Meta-analyses confirm nutrient-cancer links (41), but mechanisms lack consensus. Three gaps persist: First, research favors solid tumors (24, 42), neglecting blood cancers. Second, most studies focus on single nutrients, not dietary patterns. Third, cross-cancer analyses are methodologically limited—e.g., 30-nutrient studies on gynecological cancers (43), 15-micronutrient assessments in endometrial cancer (44), or 150-factor machine learning models in cervical cancer (45). While revealing specific associations, these lack systematic comparisons between solid and blood cancers.

In the present study, we sought to explore the association of global dietary nutrient intake with the presence of cancer, solid cancer, and blood cancers. To this end, the National Health and Nutrition Examination Survey (NHANES, 2001–2023) was utilized as a database, as it is recognized as an internationally authoritative, population-based survey. The analysis focused on the relationship between dietary nutrient intake and cancer among participating American populations. Furthermore, we sought to refine our understanding of the relationship between specific dietary nutrients and the presence of cancer, taking into account global dietary intake. A comprehensive understanding of the relationship between dietary nutrient intake and cancer, particularly solid and blood cancer, will provide a critical adjunct to subsequent cancer treatment.

## Methods

### Study population

**Figure 1:**
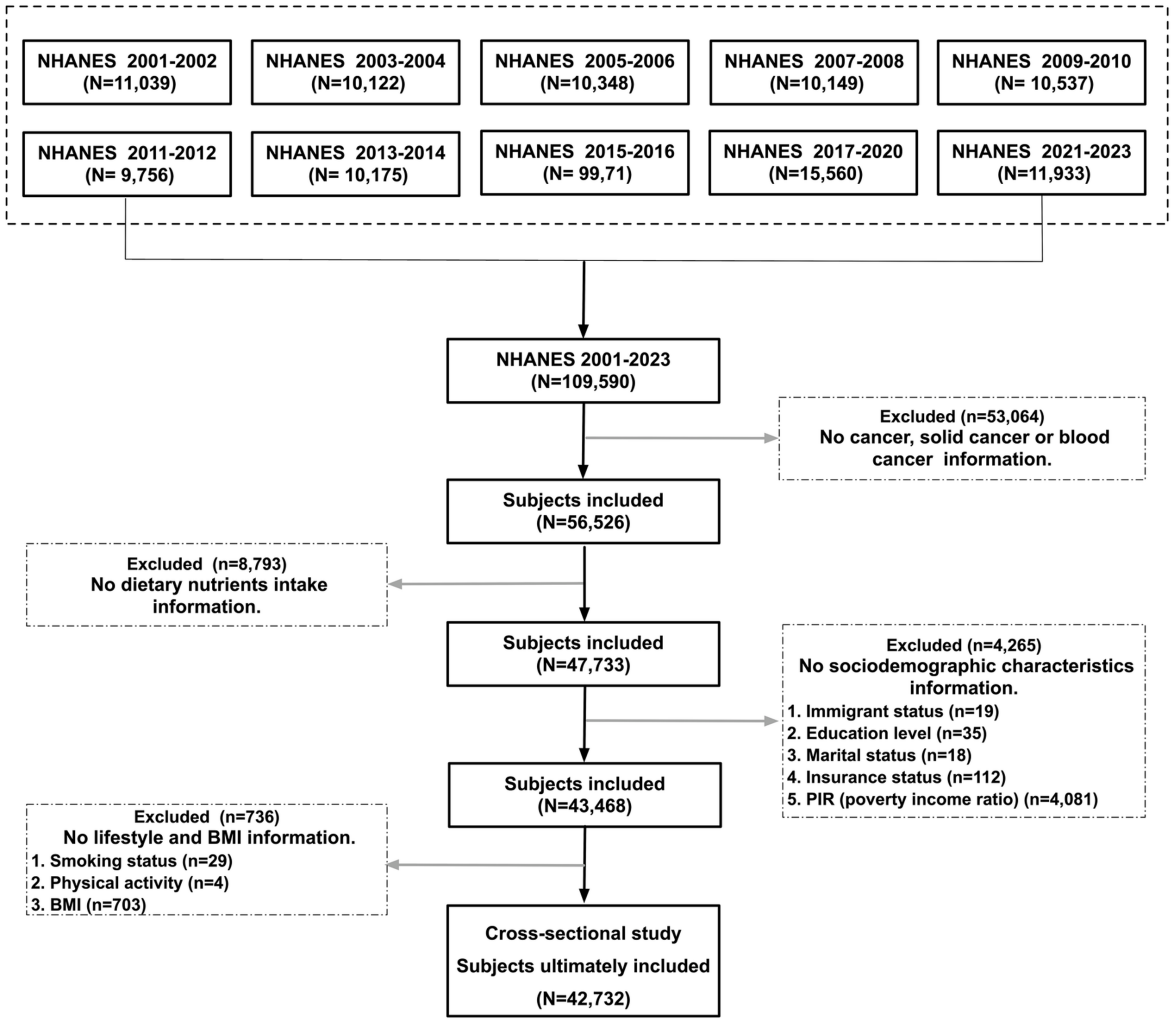
Flowchart for the inclusion of participants.

The National Health and Nutrition Examination Survey (NHANES) is a nationally representative U.S. health survey that annually collects demographic, dietary, and clinical data from approximately 10,000 mobile adults (46). For this analysis, a total of 42,732 participants (2001–2023 cycles) meeting three criteria: For this analysis, we included 42,732 participants (2001–2023 cycles) meeting three criteria: (1) age ≥ 20 years; (2) complete cancer diagnosis records and dietary assessments; (3) full covariate data [including age, sex, race, immigration status, education level, poverty-income ratio (PIR), marital status, health insurance, Smoking status, BMI, physical activity level, and total energy intake]. Individuals lacking critical information were excluded. Study reporting followed STROBE guidelines (47), with participant selection detailed in Figure 1.

### Dietary assessment

Nutrient intake was assessed using two consecutive 24-h dietary recalls from NHANES questionnaires (48). The geometric mean of both recordings was calculated to account for day-to-day variability (49). The first recall occurred in-person at Mobile Examination Centers (MEC), followed by a telephone-administered second recall (50).

Initially, the NHANES data from 2001 to 2023 was merged (comprising a total of 11 cycles). Subsequently, subjects were subjected to a multifaceted screening process, stratifying them based on their cancer-related information, dietary intake data, socio-demographic characteristics, lifestyle habits, and BMI. This comprehensive approach ultimately resulted in the enrollment of 42,732 subjects.

We analyzed 38 dietary components categorized as: Macronutrients: Protein, Carbohydrate, Total sugars, Dietary fiber, Total fat, Saturated fatty acids (SFA), Monounsaturated fatty acids (MUFA), Polyunsaturated fatty acids (PUFA), Cholesterol; Vitamins: Vitamin E, Retinol, Vitamin A, *α*-carotene, *β*-carotene, β-cryptoxanthin, Lycopene, Lutein + zeaxanthin, Thiamin (B1), Riboflavin (B2), Niacin, Folate, Vitamin B12, Ascorbic acid (C), Vitamin K; Minerals: Calcium, Phosphorus, Magnesium, Iron, Zinc, Copper, Sodium, Potassium, Selenium; Other: Caffeine, Theobromine, Moisture, Alcohol.

### Assessment of solid cancer and blood cancer

The presence of carcinoma was determined by the question: ‘Have you ever been told by a doctor or other health professional that you had cancer or a malignancy of any kind?’. The question ‘What kind of cancer was it?’ was used to determine whether the cancer was solid or blood. Participants reporting blood-related malignancies (‘Blood’, ‘Leukemia’, or ‘Lymphoma’) were classified as blood cancer cases (51). All other cancer types were categorized as solid tumors based on standardized classifications (52, 53).

### Covariates

We adjusted for covariates spanning three domains: Demographics: Age, sex, race/ethnicity (Hispanic, non-Hispanic White, non-Hispanic Black, other), and immigration status; Socioeconomic: Education level (<high school, high school, >high school), PIR (<1, 1–3, >3), marital status, and health insurance; Lifestyle/Clinical: Smoking status (never/former/current), BMI, physical activity level (sedentary/moderate/vigorous), total energy intake.

### Statistical analysis

We performed weighted logistic regression to evaluate associations between nutrient intakes and the presence of cancer calculating adjusted odds ratios (ORs) with 95% confidence intervals (CIs). NHANES dietary weights were applied to ensure national representativeness. To explore potential non-linear relationships, restricted cubic splines (RCS) with four knots were modeled for significant nutrients. Continuous variables were summarized as median (interquartile range) and categorical variables as frequency (%). Between-group comparisons used Pearson’s *χ*^2^ test for categorical data and Kruskal–Wallis test for non-normally distributed continuous variables. Analyses were conducted using SPSS 29.0 (descriptive statistics), Stata/MP 18.0 (regression modeling), and R 4.4.2 (RCS analysis), with statistical significance set at two-tailed *p* < 0.05.

## Results

### Baseline information and dietary nutrient intakes of the study participants

The baseline sociodemographic characteristics of all participants are summarized in Table 1. During the nearly 20-year period under consideration, the prevalence of cancer in the US population was 10.16% (4,342/42,732), with 9.85% (4,207/42,732) for solid cancers and 0.31% (135/42,732) for blood cancers. Compared with participants without cancer, those with cancer were more likely to be older (*p* < 0.001), to have lower energy intake (*p* < 0.001), to be female (*p* < 0.001), to be non-Hispanic White (*p* < 0.001), to live in the United States (*p* < 0.001), were more likely to be married or living with a partner (*p* = 0.012), had a higher proportion of PIR >3 (*p* < 0.001) and insurance (*p* < 0.001), were former smokers, and had moderate physical activity or sedentary habits (*p* < 0.001). These trends were almost identical for solid and blood cancers.

**Table 1:** Characteristics of participants stratified by any “Cancer Diagnosis,” “Solid Cancer Only” and “Blood Cancer Only.”

Characteristic	Any cancer diagnosis			Solid cancer only			Blood cancer only		
	Yes (*N* = 4,342)	No (*N* = 38,390)	*P*-value^2^	Yes (*N* = 4,207)	No (*N* = 38,525)	*P*-value	Yes (*N* = 135)	No (*N* = 42,597)	*P*-value
Age (years), median (IQR)^1^	65(53, 74)	44(32, 57)	**<0.001**	65(54, 74)	44(32, 57)	**<0.001**	53(40, 68)	46(33, 60)	**0.005**
BMI (kg/m^2^), median (IQR)	28(25, 33)	28(24, 33)	0.2	28(25, 33)	28(24, 33)	0.2	28(25, 32)	28(24, 33)	0.3
Energy (kcal), median (IQR)	1,842(1,462, 2,357)	1,988(1,528, 2,579)	**<0.001**	1,842(1,454, 2,347)	1,988(1,528, 2,579)	**<0.001**	1,772(1,579, 2,544)	1,974(1,520, 2,551)	0.4
Male, *n* (%)	2,021(43%)	18,485(49%)	**<0.001**	1,948(43%)	18,558(49%)	**<0.001**	73(51%)	20,433(48%)	0.6
Race			**<0.001**			**<0.001**			**0.022**
Hispanic	467(4.8%)	9,446(15%)		448(4.7%)	9,465(15%)		19(7.3%)	9,894(14%)	
Non-Hispanic White	3,084(86%)	16,749(66%)		2,996(86%)	16,837(66%)		88(80%)	19,745(68%)	
Non-Hispanic Black	567(5.2%)	8,250(12%)		547(5.1%)	8,270(12%)		20(7.8%)	8,797(11%)	
Others	224(4.0%)	3,945(7.8%)		216(4.0%)	3,953(7.7%)		8(4.5%)	4,161(7.4%)	
Immigrant (vs. Indigenous)	516(7.2%)	9,997(17%)	**<0.001**	501(7.1%)	10,012(17%)	**<0.001**	15(8.5%)	10,498(16%)	**0.068**
Education level, *n* (%)			**<0.001**			**<0.001**			0.4
<High school	801(12%)	8,743(15%)		778(12%)	8,766(15%)		23(10%)	9,521(15%)	
High school	938(21%)	8,835(24%)		904(21%)	8,869(24%)		34(23%)	9,739(24%)	
>High school	2,603(68%)	20,812(61%)		2,525(68%)	20,890(61%)		78(67%)	23,337(62%)	
PIR			**<0.001**			**<0.001**			**0.028**
<1	561(9.2%)	7,874(15%)		542(9.1%)	7,893(15%)		19(11%)	8,416(14%)	
1 to 3	1,803(33%)	15,826(36%)		1,756(33%)	15,873(36%)		47(25%)	17,582(35%)	
>3	1,978(58%)	14,690(50%)		1,909(58%)	14,759(50%)		69(63%)	16,599(50%)	
Married/Living with partner (vs. others), *n* (%)	2,593(65%)	23,010(63%)	**0.012**	2,516(66%)	23,087(63%)	**0.011**	77(63%)	25,526(63%)	>0.9
Insurance, (Yes, *n*, %)	4,113(95%)	30,449(82%)	**<0.001**	3,990(95%)	30,572(82%)	**<0.001**	123(92%)	34,439(84%)	**0.037**
Smoking, *n* (%)			**<0.001**			**<0.001**			0.4
Never	1,991(47%)	21,533(56%)		1,914(47%)	21,610(56%)		77(60%)	23,447(55%)	
Former	1,731(38%)	8,928(23%)		1,692(38%)	8,967(23%)		39(27%)	10,620(25%)	
Current	620(15%)	7,929(20%)		601(15%)	7,948(20%)		19(14%)	8,530(20%)	
Physical activity, *n* (%)			**<0.001**			**<0.001**			0.11
Sedentary	2,108(41%)	15,093(35%)		2,042(41%)	15,159(35%)		66(44%)	17,135(36%)	
Moderate	1,371(35%)	10,395(27%)		1,335(35%)	10,431(27%)		36(31%)	11,730(28%)	
Vigrous	863(24%)	12,902(38%)		830(24%)	12,935(38%)		33(25%)	13,732(36%)	

^1^Median (Q1, Q3); *n* (unweighted) (%).

^2^Design-based Kruskal–Wallis test; Pearson’s X^2: Rao and Scott adjustment.

“Any Cancer Diagnosis”: Participants with any cancer type (including both solid and blood cancers); “Solid Cancer Only”: Participants diagnosed exclusively with solid tumors; “Blood Cancer Only”: Participants diagnosed exclusively with hematologic malignancies.

Lutein and zeaxanthin (*p* < 0.001), vitamin C (*p* = 0.016), vitamin K (*p* < 0.001), caffeine (*p* < 0.001). Bold indicates statistically significant.

As illustrated in Table 2, a clear distinction emerges in the dietary nutrient intake patterns between cancer and non-cancer participants. Compared to non-cancer participants, patients with cancer consumed lower amounts of protein (*p* < 0.001), carbohydrates (*p* < 0.001), total sugars (*p* < 0.001), total fat (*p* < 0.001), SFA (*p* = 0.001), MUFA (*p* < 0.001), PUFA (*p* < 0.001), cholesterol (*p* = 0.001), lycopene (*p* < 0.001), vitamin B1 (*p* < 0.001), niacin (*p* < 0.001), vitamin B6 (*p* < 0.001), calcium (*p* < 0.001), phosphorus (*p* < 0.001), magnesium (*p* = 0.036), zinc (*p* < 0.001), sodium (*p* < 0.001), selenium (*p* < 0.001), and moisture (*p* < 0.001), whereas higher intakes of retinol (*p* < 0.001), vitamin A (*p* < 0.001), *α*-carotene (*p* < 0.001), *β*-carotene (*p* < 0.001), β-cryptoxanthin (*p* < 0.001),

**Table 2:** Baseline information on dietary nutrients intake.

Nutrient types	Cancer			Solid cancer			Blood cancer		
	Yes (*N* = 4,342)	No (*N* = 38,390)	*P*-value^1^	Yes (*N* = 4,207)	No (*N* = 38,525)	*P*-value	Yes (*N* = 135)	No (*N* = 42,597)	*P*-value
Protein (g)	71(54,91)	77(58, 101)	<0.001	71(54,91)	77(57,101)	<0.001	68(54,95)	76(57,100)	0.2
Carbohydrate (g)	218(164,278)	235(176,310)	<0.001	218(164,278)	235(176,310)	<0.001	218(181,286)	233(174,307)	0.6
Total sugars (g)	91(61,130)	98(64,143)	<0.001	91(62,129)	98(64,143)	<0.001	84(60,140)	97(64,142)	0.5
Dietary fiber (g)	16(11,21)	15(10,21)	0.2	16(11,21)	15(10,21)	0.2	15(11,19)	15(11,21)	0.6
Total fat (g)	73(52,96)	76(55,103)	<0.001	73(52,96)	76(55,103)	<0.001	75(51,99)	76(54,103)	0.7
SFA (g)	23(17,32)	24(17,34)	0.001	23(17,32)	24(17,34)	0.001	22(16,34)	24(17,34)	0.6
MUFA (g)	25(18,34)	27(19,37)	<0.001	25(18,34)	27(19,37)	<0.001	26(18,36)	26(19,37)	0.8
PUFA (g)	16(11,22)	17(11,23)	0.017	16(11,22)	17(11,23)	0.013	18(11,24)	17(11,23)	0.8
Cholesterol (mg)	234(145,363)	248(156,385)	0.001	234(145,362)	248(155,385)	0.001	229(128,401)	247(155,383)	0.3
Vitamin E (mg)	7.2(5.1,10.4)	7.2(4.9,10.5)	0.7	7.2(5.1,10.4)	7.2(4.9,10.5)	0.6	6.9(4.7,10.3)	7.2(4.9,10.5)	0.5
Retinol (μg)	370(224,559)	342(200,547)	<0.001	371(224,560)	342(200,546)	<0.001	337(205,542)	345(202,548)	0.8
Vitamin A (μg)	567(369,849)	524(321,805)	<0.001	568(373,852)	524(321,805)	<0.001	531(275,750)	529(325,810)	0.6
α-carotene (μg)	115(29,543)	73(21,422)	<0.001	117(29,546)	73(21,422)	<0.001	63(16,262)	76(22,433)	0.2
β-carotene (μg)	1,409(534,3,287)	1,058(427,2,734)	<0.001	1,422(542,3,342)	1,056(427,2,733)	<0.001	969(327,2,400)	1,093(435,2,790)	0.3
β-cryptoxanthin (μg)	44(16,112)	38(13,102)	<0.001	45(16,112)	38(13,102)	<0.001	33(8,100)	39(13,103)	0.5
Lycopene (μg)	2,149(517,6,100)	2,585(660,6,882)	<0.001	2,125(515,6,129)	2,585(660,6,875)	<0.001	2,351(839,5,569)	2,544(638,6,796)	0.7
Lutein and zeaxanthin (μg)	970(515,1,875)	832(443,1,623)	<0.001	971(518,1,875)	832(443,1,623)	<0.001	907(440,1,775)	847(449,1,650)	>0.9
Vitamin B1 (mg)	1.42(1.06,1.86)	1.47(1.08,1.98)	<0.001	1.42(1.06,1.85)	1.47(1.08,1.98)	<0.001	1.53(1.04,1.97)	1.47(1.08,1.97)	0.8
Vitamin B2 (mg)	1.93(1.45,2.54)	1.94(1.40,2.63)	0.5	1.93(1.45,2.54)	1.94(1.40,2.63)	0.6	1.83(1.37,2.53)	1.94(1.41,2.61)	0.6
Niacin (mg)	21(16,28)	23(17,31)	<0.001	21(16,28)	23(17,31)	<0.001	23(18,30)	23(17,31)	>0.9
Vitamin B6 (mg)	1.71(1.25,2.34)	1.81(1.28,2.52)	<0.001	1.71(1.25,2.35)	1.81(1.28,2.52)	<0.001	1.63(1.27,2.29)	1.80(1.28,2.50)	0.3
Food folate (μg)	193(142,267)	197(138,272)	0.5	193(142,268)	197(138,272)	0.6	196(133,247)	196(139,272)	0.5
Vitamin B12 (μg)	4.0(2.6,6.0)	4.0(2.5,6.3)	0.11	4.0(2.6,6.0)	4.0(2.5,6.3)	0.087	4.1(2.5,6.1)	4.0(2.5,6.3)	0.7
Vitamin C (mg)	67(32,112)	61(28,114)	0.016	67(32,113)	61(28,114)	0.012	64(32,103)	61(29,114)	0.9
Vitamin K (μg)	83(49,141)	74(43,130)	<0.001	83(49,141)	74(43,130)	<0.001	68(45,114)	75(44,131)	0.5
Calcium (mg)	819(585,1,107)	851(591,1,192)	<0.001	819(587,1,108)	851(591,1,192)	<0.001	810(511,1,027)	848(590,1,185)	0.10
Phosphorus (mg)	1,226(932,1,533)	1,276(962,1,668)	<0.001	1,229(933,1,533)	1,276(962,1,667)	<0.001	1,154(929,1,538)	1,271(959,1,651)	0.2
Magnesium (mg)	274(206,351)	278(208,365)	0.036	274(205,353)	278(208,365)	0.036	272(221,335)	278(208,364)	0.9
Iron (mg)	13(10,18)	13(10,18)	0.018	13(10,18)	13(10,18)	0.014	12(11,19)	13(10,18)	0.7
Zinc (mg)	9.7(7.3,13.2)	10.3(7.3,14.1)	<0.001	9.7(7.2,13.2)	10.3(7.3,14.1)	<0.001	9.6(7.5,13.4)	10.2(7.3,14.0)	0.5
Copper (mg)	1.13(0.85,1.49)	1.14(0.85,1.53)	0.12	1.12(0.85,1.49)	1.14(0.85,1.53)	0.093	1.19(0.94,1.45)	1.14(0.85,1.53)	0.6
Sodium (mg)	2,978(2,282,3,902)	3,238(2,409,4,264)	<0.001	2,979(2,282,3,899)	3,238(2,409,4,263)	<0.001	2,872(2,280,4,053)	3,209(2,394,4,224)	0.2
Potassium (mg)	2,547(1,938,3,208)	2,515(1,891,3,259)	0.5	2,552(1,938,3,202)	2,515(1,891,3,260)	0.4	2,414(1,784,3,351)	2,520(1,897,3,252)	0.8
Selenium (μg)	96(72,126)	105(76,140)	<0.001	96(72,126)	104(76,140)	<0.001	96(77,133)	104(76,138)	0.4
Caffeine (mg)	144(51,249)	117(38,232)	<0.001	145(50,249)	117(38,232)	<0.001	129(56,222)	120(39,235)	0.4
Theobromine (mg)	15(0,50)	13(0,49)	0.004	15(0,49)	13(0,49)	0.005	14(0,61)	13(0,49)	0.4
Alcohol (g)	0(0,8)	0(0,9)	0.6	0(0,8)	0(0,9)	0.6	0(0,13)	0(0,9)	0.7
Moisture (g)	2,577(1,935,3,317)	2,673(1,981,3,560)	<0.001	2,575(1,935,3,317)	2,672(1,981,3,559)	<0.001	2,650(1,851,3,281)	2,661(1,974,3,535)	0.4

^1^Design-based Kruskal–Wallis test. SFA, saturated fatty acid; MUFA, monounsaturated fatty acid; PUFA, polyunsaturated fatty acid and theobromine (*p* = 0.004).

### Associations between dietary nutrient intake and cancer presence

Cross-sectional associations between 38 dietary nutrient intakes and cancer are presented in Table 3 and Supplementary Table S1. Following adjustment for all potential confounding variables, significant positive associations were observed for intakes of SFA [OR, 95% CI; 1.1082(1.0110, 1.2146), *p* = 0.028], β-carotene [1.0431(1.0096, 1.0777), *p* = 0.011], and vitamin K [1.0370(1.0094, 1.0654), *p* = 0.008] with the presence of cancer, whereas phosphorus intake [0.9016(0.8218, 0.9892), *p* = 0.029] was negatively associated with cancer presence. In addition, data analysis showed that intakes of SFA [1.1099(1.0113, 1.2180), *p* = 0.029], *α*-carotene [1.0353(1.0033, 1.0683), *p* = 0.030], β-carotene [1.0484(1.0146, 1.0833), *p* = 0.005], and vitamin K [1.0405(1.0098, 1.0722), *p* = 0.009] were all positively associated with the presence of solid cancers. For blood cancer, retinol intake [1.0935(1.0222, 1.1698), *p* = 0.009] demonstrated a positive association with the presence of blood cancer. These associations between specific nutrient intakes and the presence of cancer were also confirmed in the non-standardized data (Supplementary Table S1). There were no statistically significant associations between intake of other dietary nutrients and the presence of cancers, solid tumors and blood cancers (*p* > 0.05).

**Table 3:** Odds ratios (ORs) and 95% CIs of standardized dietary nutrient intakes associated with the presence of cancer, solid cancer, and blood cancer.

Nutrient types	Cancer OR (Cl)	*p*-value	Solid cancer OR (Cl)	*p*-value	Blood cancer OR (Cl)	*p*-value
Protein (g)	0.9275(0.8446,1.0186)	0.115	0.9374(0.8520,1.0314)	0.185	0.7433(0.5078,1.0882)	0.127
Carbohydrate (g)	0.9593(0.8567,1.0741)	0.471	0.9499(0.8463,1.0662)	0.383	1.2030(0.7564,1.9133)	0.435
Total sugars (g)	1.0102(0.9379,1.0880)	0.789	1.0091(0.9353,1.0887)	0.815	1.0303(0.7618,1.3933)	0.847
Dietary fiber (g)	0.9988(0.9370,1.0648)	0.972	1.0070(0.9436,1.0747)	0.833	0.8047(0.6092,1.0630)	0.126
Total fat (g)	1.0870(0.9971,1.2168)	0.147	1.0881(0.9700,1.2205)	0.150	1.0392(0.6090,1.7735)	0.888
SFA (g)	**1.1082(1.0110,1.2146)**	**0.028** ^*^	**1.1099(1.0113,1.2180)**	**0.029** ^*^	1.0319(0.6492,1.6404)	0.894
MUFA (g)	1.0460(0.9523,1.1490)	0.347	1.0457(0.9502,1.1509)	0.360	1.0470(0.6901,1.5885)	0.829
PUFA (g)	1.0078(0.9340,1.0875)	0.840	1.0071(0.9317,1.0886)	0.858	1.0238(0.7618,1.3759)	0.876
Cholesterol (mg)	1.0284(0.9643,1.0968)	0.394	1.0334(0.9678,1.1034)	0.326	0.9088(0.6829,1.2093)	0.512
Vitamin E (mg)	0.9864(0.9333,1.0425)	0.627	0.9920(0.9379,1.0493)	0.779	0.8434(0.6471,1.0992)	0.208
Retinol (μg)	0.9845(0.9319,1.0402)	0.578	0.9679(0.9166,1.0220)	0.240	**1.0935(1.0222,1.1698)**	**0.009** ^**^
Vitamin A (μg)	1.0261(0.9714,1.0839)	0.356	1.0214(0.9653,1.0808)	0.463	1.0758(0.9525,1.2151)	0.239
α-carotene (μg)	1.0310(0.9974,1.0658)	0.071	**1.0353(1.0033,1.0683)**	**0.030** ^*^	0.7089(0.4567,1.1004)	0.125
β-carotene (μg)	**1.0431(1.0096,1.0777)**	**0.011** ^*^	**1.0484(1.0146,1.0833)**	**0.005** ^**^	0.7594(0.5479,1.0524)	0.098
β-cryptoxanthin (μg)	0.9559(0.9037,1.0111)	0.115	0.9617(0.9094,1.0170)	0.171	0.7553(0.5481,1.0408)	0.086
Lycopene (μg)	0.9533(0.9052,1.0039)	0.070	0.9574(0.9083,1.0091)	0.105	0.8524(0.6860,1.0592)	0.150
Lutein and zeaxanthin (μg)	1.0346(0.9932,1.0778)	0.102	1.0375(0.9964,1.0803)	0.074	0.9177(0.7260,1.1601)	0.473
Vitamin B1 (mg)	0.9874(0.9219,1.0575)	0.717	0.9800(0.9138,1.0510)	0.572	1.1451(0.8875,1.4773)	0.297
Vitamin B2 (mg)	0.9516(0.8749,1.0350)	0.247	0.9544(0.8762,1.0396)	0.285	0.9099(0.6163,1.3435)	0.635
Niacin (mg)	0.9349(0.8514,1.0267)	0.159	0.9327(0.8466,1.0277)	0.159	0.9945(0.8177,1.2096)	0.956
Vitamin B6 (mg)	0.9747(0.9051,1.0497)	0.498	0.9804(0.9093,1.0569)	0.605	0.8363(0.6359,1.0998)	0.201
Food folate (μg)	1.0129(0.9413,1.0900)	0.731	1.0191(0.9469,1.0967)	0.614	0.8615(0.6194,1.1984)	0.376
Vitamin B12 (μg)	0.9860(0.9365,1.0381)	0.592	0.9800(0.9297,1.0332)	0.454	1.0672(0.9801,1.1621)	0.134
Vitamin C (mg)	0.9871(0.9274,1.0506)	0.682	0.9901(0.9289,1.0552)	0.759	0.9093(0.7414,1.1152)	0.361
Vitamin K (μg)	**1.0370(1.0094,1.0654)**	**0.008** ^**^	**1.0405(1.0098,1.0722)**	**0.009** ^**^	0.8206(0.5784,1.1641)	0.268
Calcium (mg)	0.9550(0.8953,1.0187)	0.162	0.9626(0.9015,1.0279)	0.255	0.7865(0.5720,1.0815)	0.140
Phosphorus (mg)	**0.9016(0.8218,0.9892)**	**0.029** ^*^	0.9106(0.8287,1.0006)	0.052	0.7325(0.4717,1.1373)	0.165
Magnesium (mg)	0.9668(0.8967,1.0424)	0.380	0.9684(0.8967,1.0459)	0.414	0.9613(0.7214,1.2811)	0.788
Iron (mg)	1.0080(0.9427,1.0778)	0.817	1.0046(0.9377,1.0763)	0.896	1.0836(0.8808,1.3331)	0.448
Zinc (mg)	0.9606(0.9057,1.0188)	0.181	0.9632(0.9079,1.0219)	0.214	0.8980(0.6148,1.3118)	0.578
Copper (mg)	0.9807(0.9283,1.0360)	0.486	0.9754(0.9216,1.0323)	0.388	1.0582(0.9850,1.1367)	0.122
Sodium (mg)	1.0067(0.9197,1.1019)	0.885	1.0168(0.9279,1.1143)	0.721	0.7915(0.5025,1.2466)	0.313
Potassium (mg)	0.9815(0.9014,1.0687)	0.667	0.9849(0.9028,1.0744)	0.731	0.9255(0.6852,1.2502)	0.614
Selenium (μg)	0.9468(0.8746,1.0249)	0.176	0.9459(0.8723,1.0257)	0.178	0.9822(0.7047,1.3691)	0.916
Caffeine (mg)	0.9731(0.9243,1.0245)	0.299	0.9700(0.9218,1.0208)	0.242	1.0909(0.7996,1.4884)	0.583
Theobromine (mg)	0.9957(0.9484,1.0453)	0.861	0.9903(0.9418,1.0413)	0.704	1.1064(0.9791,1.2503)	0.105
Alcohol (g)	1.0024(0.9435,1.0651)	0.937	1.0060(0.9458,1.0700)	0.850	0.9175(0.7147,1.1777)	0.499
Moisture (g)	1.0282(0.9711,1.0886)	0.340	1.0346(0.9760,1.0966)	0.253	0.9140(0.7153,1.1679)	0.472

ORs, odd ratios; CIs, confidence interval; *p < 0.05, **p < 0.01, ***p < 0.001. Bold indicates statistically significant.

**Figure 2:**
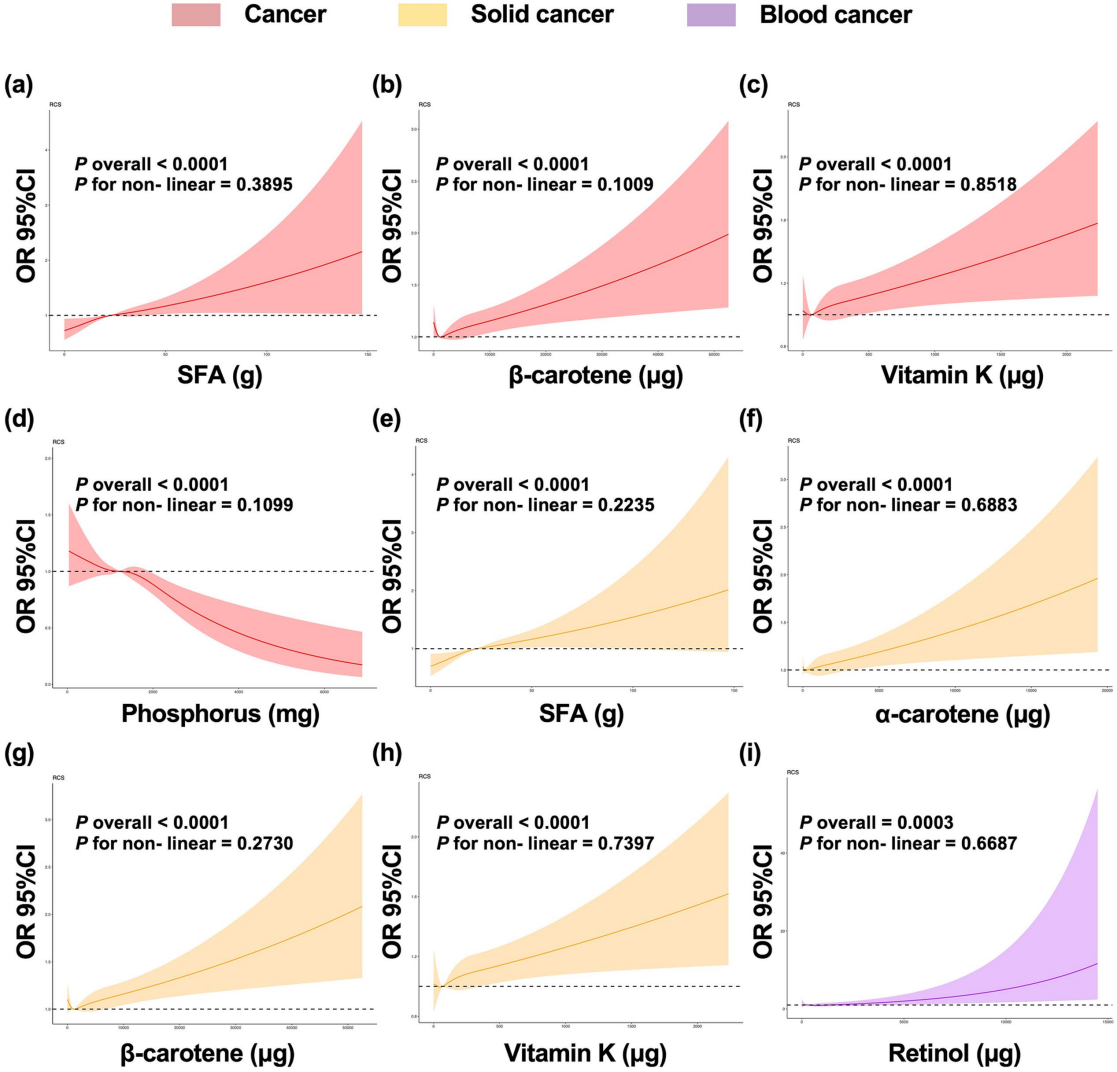
Dose–response curves between intake of candidate nutrients and the presence of cancer, solid cancer, and blood cancer. **(a–i)** The association between specific nutrient intake and cancer presence, subsequent to adjustment for all covariates. OR, odd ratio; 95% CI, confidence interval; SFA, saturated fatty acid.

To further investigate the relationship between specific nutrient intake and cancer presence, curve fitting was performed after adjustment for all possible confounding factors. Figure 2 shows the trends of cancer, solid cancer and blood cancer presences with the intake of specific candidate nutrients according to the results of the correlation analyses. The intakes of SFA (cancer: *p* overall <0.0001, *p* for non-linear = 0.3895; solid cancer: *p* overall <0.0001, *p* for non-linear = 0.2235), *β*-carotene (*p* overall <0.0001, *p* for non-linear = 0.1009; *p* overall <0.0001, *p* for non-linear = 0.2730), and vitamin K (*p* overall <0.0001, *p* for non-linear = 0.8518; *p* overall <0.0001, *p* for non-linear = 0.7397) were overall positively associated with the presence of cancer and solid cancer, and no non-linear association was observed. Phosphorus intake (*p* overall <0.0001, *p* for non-linear = 0.1099) is negatively associated with cancer presence. Additionally, *α*-carotene intake (*p* overall <0.0001, *p* for non-linear = 0.6883) was positively associated with the presence of solid cancers, and retinol intake (*p* overall = 0.0003, *p* for non-linear = 0.6687) was also positively linked with the presence of blood cancers.

## Discussion

This study aimed to systematically evaluate associations between 38 dietary nutrients and cancer presence, with particular focus on differential effects in solid tumors versus blood cancers. By analyzing comprehensive NHANES data, we sought to overcome limitations of prior single-nutrient or single-cancer-type studies, thereby identifying potential pan-cancer dietary risk modifiers. Our study provides novel evidence linking six dietary components—saturated fatty acids (SFA), retinol, *α*-carotene, *β*-carotene, vitamin K, and phosphorus—to cancer presence when analyzed through a comprehensive nutrient intake framework. Dose–response analyses revealed positive associations between elevated SFA, retinol, carotenoids, and vitamin K with cancer presence, while adequate phosphorus intake exerted protective associations. To our knowledge, this represents the first population-level investigation evaluating integrated dietary patterns across both solid and blood cancers.

Existing literature primarily examines isolated nutrients. Recent cohort data associate high SFA intake with colorectal cancer (54), corroborated by meta-analyses linking SFA to breast, prostate, and colorectal malignancies (20)—aligning with our observed SFA-cancer associations. Intriguingly, while meta-analyses suggest *β*-carotene inversely correlates with a reduced presence of breast cancer (55), null associations emerge for gastric cancer (56). Our findings contrast by demonstrating positive *α*/β-carotene-cancer links, paralleling a Singaporean case–control study showing elevated serum carotenoids with prostate cancer (57). These discrepancies likely stem from: (1) Cancer type specificity: Site-specific vs. pan-cancer analyses; (2) Exposure assessment: Serum biomarkers vs. dietary intake quantification; (3) Study design limitations in prior single-nutrient approaches. Resolving these contradictions requires large-scale RCTs integrating multi-omic biomarkers with longitudinal dietary monitoring.

Our analysis further identifies vitamin K intake as positively associated with both overall and solid cancer presence—a finding that contrasts with observational studies suggesting anti-cancer benefits of vitamin K supplementation (32, 58–61). Notably, a U.S. cohort study may align with our findings, reporting increased breast cancer incidence and mortality with higher vitamin K intake (62). This paradox may stem from: (1) source differentiation: Supplemental vs. dietary vitamin K forms (phylloquinone vs. menaquinones); (2) Cancer stage specificity: chemoprotective effects in early carcinogenesis vs. pro-tumor impacts in established malignancies. Regarding phosphorus, we observed protective associations at moderate intake levels, consistent with Zhu et al.’s (43) gynecological cancer findings. Preclinical studies further support phosphorus derivatives as promising anticancer nanocarriers (63), though epidemiological evidence remains conflicting (64, 65). The inverse association between phosphorus and cancer mirrors preclinical evidence of phosphate restriction slowing tumor growth (39, 66), suggesting a therapeutic avenue for dietary modulation. Mechanistic studies should clarify whether phosphorus exerts direct antineoplastic effects or serves as a biomarker for calcium-phosphate homeostasis.

The complex interplay between dietary nutrients and cancer likely operates through multiple synergistic biological pathways. Antioxidant nutrients such as vitamin C, vitamin E, and *β*-carotene exert protective effects by neutralizing reactive oxygen species (ROS) and reducing oxidative DNA damage—a hallmark of carcinogenesis (67–69). Selenium complements this defense by enhancing glutathione peroxidase activity, mitigating lipid peroxidation and genomic instability (70). However, pro-inflammatory dietary components like saturated fats may counteract these benefits by activating NF-κB signaling, which upregulates angiogenic cytokines (e.g., IL-6, TNF-*α*) to fuel tumor progression (71). Beyond direct oxidative mechanisms, nutrients modulate epigenetic landscapes: folate regulates DNA methylation patterns critical for tumor suppressor gene expression (72), while vitamin D induces cell cycle arrest through vitamin D receptor (VDR) activation (73). Dietary fibers further contribute to cancer prevention via gut microbiota-derived metabolites like butyrate, which selectively induce apoptosis in precancerous colonic cells (74, 75).

At the metabolic level, high saturated fat intake has been shown to epigenetically reprogram oncogenic pathways and activate pathological lipid metabolism in preclinical models (54). Vitamin K’s dual roles—modulating oxidative stress and regulating apoptosis through steroid/xenobiotic receptors—may explain its context-dependent associations with cancer outcomes (33, 76). Phosphorus’s potential anticancer effects, possibly mediated through redox balance restoration in the tumor microenvironment, remain mechanistically elusive but clinically suggestive (63). The paradoxical associations of carotenoids (*α*/*β*-carotene) with cancer could stem from their biphasic effects on inflammatory signaling, warranting single-cell resolution studies to delineate tissue-specific impacts (77–79). These mechanisms converge on cancer’s metabolic vulnerabilities. Emerging evidence suggests that strategic nutritional modulation—such as retinol’s regulation of ferroptosis (80) or phosphorus-mediated redox modulation—could enhance conventional chemotherapy by disrupting tumor metabolic dependencies (81, 82). Nevertheless, definitive causal attribution requires innovative models, particularly patient-derived organoids, to isolate nutrient effects from confounding lifestyle variables.

Furthermore, it is critical to recognize that cancer and its treatments may reciprocally alter nutrient intake patterns. Chemotherapy and radiation commonly induce anorexia, taste alterations (e.g., dysgeusia), and gastrointestinal toxicities (e.g., mucositis), significantly reducing dietary diversity and calorie consumption (83, 84). For instance, majority of patients report chemotherapy-induced taste changes that persist beyond treatment, preferentially reducing protein and vegetable intake (85, 86). Additionally, malignancies like pancreatic or gastrointestinal cancers directly impair nutrient absorption through mechanical obstruction or metabolic dysfunction (87, 88). These treatment- and disease-driven nutritional deficits may partly explain the lower macronutrient intake observed in cancer patients (Table 2). Recent clinical guidelines emphasize proactive nutritional support to mitigate these effects, highlighting the need for longitudinal studies disentangling causative dietary influences from treatment sequelae.

While this study identifies significant associations between nutrient profiles and cancer presence, several limitations warrant cautious interpretation. A primary constraint stems from the cross-sectional design, where dietary data collection occurred post-diagnosis. Cancer therapies—including chemotherapy and radiation—often reduce appetite and alter taste perception, likely contributing to observed nutritional disparities (e.g., lower macronutrient intake in cancer patients) rather than reflecting pre-disease dietary habits. Furthermore, self-reported cancer histories may introduce recall bias, and while NHANES protocols ensure methodological rigor, two 24-h dietary recalls might inadequately represent long-term consumption patterns. Critically, the temporal ambiguity inherent to observational designs prevents distinguishing whether dietary patterns influence cancer development or result from disease progression. Unaccounted interactions between nutrients and therapies further complicate causal attribution.

Despite these limitations, three key strengths bolster the findings’ validity: (1) Multivariable adjustments minimized confounding by sociodemographic, lifestyle, and energy intake variables; (2) restricted cubic spline analyses revealed non-linear dose–response relationships between specific nutrients (e.g., vitamin K, *β*-carotene) and cancer presence; (3) the nationally representative NHANES cohort (*N* = 42,732) offers robust statistical power and relevance to contemporary U.S. dietary practices. Future research should prioritize longitudinal designs with pre-diagnosis dietary assessments and clinical trials targeting nutrients showing threshold effects (e.g., vitamin K reduction trials), which could clarify causality and therapeutic applications. By evaluating 38 nutrients across cancer types, this study advances beyond reductionist approaches to reveal context-dependent dietary associations. The distinct associations for solid vs. blood cancers (e.g., retinol’s hematologic specificity) underscore the need for precision nutrition strategies tailored to cancer biology.

## Conclusion

Our findings reveal significant nutrient-cancer associations, though the causal direction remains unclear due to potential treatment-induced dietary changes. While these patterns highlight promising targets for nutritional interventions, their clinical translation requires rigorous validation through multi-center longitudinal studies tracking pre-diagnosis diets across diverse populations. Priority should be given to randomized trials testing therapeutic modulation of threshold-effect nutrients (e.g., vitamin K reduction) before integrating such strategies into adjuvant therapies. This evidence hierarchy will determine whether observed associations reflect modifiable risk factors or secondary disease manifestations.

## Data Availability

The datasets presented in this study can be found in online repositories. The names of the repository/repositories and accession number(s) can be found: NHANES repository (https://www.cdc.gov/nchs/nhanes/index.htm).
