# Supplementary material for: Dietary nutrient intake and cancer presence: evidence from a cross-sectional study
Source: Front Nutr. 2025 Apr 1;12:1551822. doi: 10.3389/fnut.2025.1551822 (PMC11996664; doi:10.3389/fnut.2025.1551822)
Supplement: Supplementary file 1 [file Data_Sheet_1.zip › Supplementary material/Table S1.docx]

**Table S1.** ORs and 95% CIs for the associations of untreated dietary nutrient intakes with cancer, solid cancer, and blood cancer.

| **Nutrient types** | **Cancer OR (Cl)** | ***p*-Value** | **Solid cancer OR (Cl)** | ***p*-Value** | **Blood cancer OR (Cl)** | ***p*-Value** |
| --- | --- | --- | --- | --- | --- | --- |
| Protein (g) | 0.9980(0.9954,1.0005) | 0.115 | 0.9983(0.9957,1.0008) | 0.185 | 0.9920(0.9818,1.0023) | 0.127 |
| Carbohydrate (g) | 0.9996(0.9986,1.0006) | 0.471 | 0.9995(0.9985,1.0006) | 0.383 | 1.0016(0.9975,1.0058) | 0.435 |
| Total sugars (g) | 1.0001(0.9991,1.0012) | 0.789 | 1.0001(0.9990,1.0012) | 0.815 | 1.0004(0.9961,1.0048) | 0.847 |
| Dietary fiber (g) | 0.9999(0.9930,1.0068) | 0.972 | 1.0008(0.9938,1.0078) | 0.833 | 0.9769(0.9481,1.0066) | 0.126 |
| Total fat (g) | 1.0020(0.9993,1.0048) | 0.148 | 1.0021(0.9993,1.0049) | 0.150 | 1.0009(0.9980,1.0141) | 0.888 |
| SFA (g) | **1.0070(1.0008,1.0134)** | **0.028^*^** | **1.0071(1.0007,1.0135)** | **0.029^*^** | 1.0022(0.9709,1.0344) | 0.894 |
| MUFA (g) | 1.0029(0.9968,1.0090) | 0.347 | 1.0029(0.9967,1.0091) | 0.360 | 1.0030(0.9763,1.0304) | 0.829 |
| PUFA (g) | 1.0007(0.9936,1.0079) | 0.840 | 1.0007(0.9934,1.0080) | 0.858 | 1.0022(0.9748,1.0304) | 0.876 |
| Cholesterol (mg) | 1.0001(0.9998,1.0004) | 0.394 | 1.0002(0.9998,1.0005) | 0.326 | 1.0095(0.9982,1.0009) | 0.512 |
| Vitamin E (mg) | 0.9975(0.9877,1.0075) | 0.627 | 0.9986(0.9985,1.0087) | 0.779 | 0.9698(0.9248,1.0171) | 0.208 |
| Retinol (μg) | 1.0000(0.9999,1.0001) | 0.578 | 0.9999(0.9998,1.0000) | 0.240 | **1.0002(1.0000,1.0003)** | **0.009^**^** |
| Vitamin A (μg) | 1.0001(1.0000,1.0001) | 0.356 | 1.0000(0.9999,1.0001) | 0.463 | 1.0001(0.9999,1.0003) | 0.239 |
| α-carotene (μg) | 1.0000(1.0000,1.0001) | 0.071 | **1.0000(1.0000,1.0001)** | **0.030^*^** | 0.9997(0.9993,1.0001) | 0.125 |
| β-carotene (μg) | **1.0001(1.0000,1.0002)** | **0.011^*^** | **1.0000(1.0000,1.0001)** | **0.005^**^** | 0.9999(0.9998,1.0000) | 0.098 |
| β--cryptoxanthin (μg) | 0.9998(0.9996,1.0000) | 0.115 | 0.9998(0.9996,1.0001) | 0.171 | 0.9988(0.9974,1.0002) | 0.086 |
| Lycopene (μg) | 1.0000(1.0000,1.0000) | 0.070 | 1.0000(1.0000,1.0000) | 0.105 | 1.0000(1.0000,1.0000) | 0.150 |
| Lutein&zeaxanthin (μg) | 1.0000(1.0000,1.0000) | 0.102 | 1.0000(1.0000,1.0000) | 0.074 | 1.0000(0.9999,1.0001) | 0.473 |
| Vitamin B1(mg) | 0.9843(0.9037,1.0721) | 0.717 | 0.9752(0.8938,1.0639) | 0.572 | 1.1838(0.8619,1.6258) | 0.297 |
| Vitamin B2 (mg) | 0.9573(0.8892,1.0307) | 0.247 | 0.9598(0.8903,1.0347) | 0.285 | 0.9204(0.6535,1.2963) | 0.635 |
| Niacin (mg) | 0.9950(0.9881,1.0020) | 0.159 | 0.9948(0.9876,1.0020) | 0.159 | 0.9996(0.9851,1.0143) | 0.956 |
| Vitamin B6 (mg) | 0.9816(0.9301,1.0359) | 0.498 | 0.9857(0.9333,1.0411) | 0.605 | 0.8782(0.7197,1.0716) | 0.201 |
| Food folate (μg) | 1.0001(0.9995,1.0007) | 0.731 | 1.0002(0.9996,1.0007) | 0.614 | 0.9988(0.9962,1.0014) | 0.376 |
| Vitamin B12 (μg) | 0.9977(0.9893,1.0062) | 0.592 | 0.9967(0.9881,1.0054) | 0.454 | 1.0108(0.9967,1.0250) | 0.134 |
| Vitamin C (mg) | 0.9998(0.9991,1.0006) | 0.682 | 0.9999(0.9991,1.0006) | 0.759 | 0.9989(0.9964,1.0013) | 0.361 |
| Vitamin K (μg) | **1.0002(1.0001,1.0004)** | **0.008^**^** | **1.0002(1.0001,1.0004)** | **0.009^**^** | 0.9988(0.9968,1.0009) | 0.268 |
| Calcium (mg) | 0.9999(0.9998,1.0000) | 0.162 | 0.9999(0.9998,1.0001) | 0.255 | 0.9995(0.9989,1.0002) | 0.140 |
| Phosphorus (mg) | **0.9998(0.9997,1.0000)** | **0.029^*^** | 0.9998(0.9997,1.0000) | 0.052 | 0.9995(0.9987,1.0002) | 0.165 |
| Magnesium (mg) | 0.9997(0.9992,1.0003) | 0.380 | 0.9998(0.9992,1.0003) | 0.414 | 0.9997(0.9976,1.0018) | 0.788 |
| Iron (mg) | 1.0010(0.9924,1.0098) | 0.817 | 1.0006(0.9917,1.0096) | 0.896 | 1.0105(0.9837,1.0380) | 0.448 |
| Zinc (mg) | 0.9943(0.9860,1.0027) | 0.181 | 0.9947(0.9863,1.0031) | 0.214 | 0.9848(0.9330,1.0394) | 0.578 |
| Copper (mg) | 0.9807(0.9282,1.0361) | 0.486 | 0.9753(0.9214,1.0323) | 0.388 | 1.0583(0.9850,1.1370) | 0.122 |
| Sodium (mg) | 1.0000(0.9999,1.0001) | 0.885 | 1.0000(1.0000,1.0001) | 0.721 | 0.9999(0.9996,1.0001) | 0.313 |
| Potassium (mg) | 1.0000(0.9999,1.0001) | 0.667 | 1.0000(0.9999,1.0001) | 0.731 | 0.9999(0.9997,1.0002) | 0.614 |
| Selenium (μg) | 0.9990(0.9976,1.0004) | 0.176 | 0.9990(0.9976,1.0005) | 0.178 | 0.9997(0.9937,1.0057) | 0.916 |
| Caffeine (mg) | 0.9998(0.9996,1.0001) | 0.299 | 0.9998(0.9995,1.0001) | 0.242 | 1.0005(0.9987,1.0022) | 0.583 |
| Theobromine (mg) | 0.9999(0.9991,1.0007) | 0.861 | 0.9998(0.9990,1.0007) | 0.704 | 1.0016(0.9997,1.0036) | 0.105 |
| Alcohol (g) | 1.0001(0.9975,1.0027) | 0.937 | 1.0003(0.9976,1.0029) | 0.850 | 0.9963(0.9856,1.0071) | 0.499 |
| Moisture (g) | 1.0000(1.0000,1.0001) | 0.340 | 1.0000(1.0000,1.0001) | 0.253 | 0.9999 (0.9997,1.0001) | 0.472 |

ORs, odd ratios; CIs, confidence interval; * *p*<0.05, ** *p*<0.01, *** *p*<0.001
