# Supplementary material for: Dietary nutrient intake and cancer presence: evidence from a cross-sectional study
Source: Front Nutr. 2025 Apr 1;12:1551822. doi: 10.3389/fnut.2025.1551822 (PMC11996664; doi:10.3389/fnut.2025.1551822)
Supplement: Supplementary file 1 [file Data_Sheet_1.zip › Supplementary material/Supplementary Figure and Table legends.docx]

**Supplementary Table legends**

**Table S1. ORs and 95% CIs for the associations of untreated dietary nutrient intakes with cancer, solid cancer, and blood cancer.**

ORs, odd ratios; CI, confidence interval; * *p*<0.05, ** *p*<0.01, *** *p*<0.001
